# Anatomical repair of a bilateral Tessier No. 3 cleft by midfacial advancement

**DOI:** 10.1186/s40902-018-0147-3

**Published:** 2018-05-05

**Authors:** Ji-hyeon Oh, Young-Wook Park

**Affiliations:** Department of Oral and Maxillofacial Surgery, College of Dentistry, Gangneung-Wonju National University, 7, Jukheon-gil, Gangneung-si, Gangwon-do South Korea

**Keywords:** Bilateral Tessier 3, Anatomic repair, Midfacial advancement, Facial muscle reposition, Anophthalmos

## Abstract

**Background:**

Bilateral Tessier number 3 clefts are extremely rare, and their surgical treatments have not been well established.

**Case presentation:**

The authors describe the case of a patient with a right Tessier number 3, 11 facial cleft with microphthalmia, a left Tessier number 3 facial cleft with anophthalmia, and cleft palate. We repaired simultaneously the bilateral soft tissue clefts by premaxillary repositioning, cleft lip repair, facial cleft repair by nasal lengthening, midfacial advancement, and an upper eyelid transposition flap with repositioning both the medial canthi. Postoperatively, the patient showed an esthetically acceptable face without unnatural scars.

**Conclusions:**

We achieved good results functionally and esthetically by midfacial advancement with facial muscle reposition instead of traditional interdigitating Z-plasties. The surgical modality of our anatomical repair and 3 months follow-up results are presented.

## Background

The Tessier number (No.) 3 cleft is an uncommon congenital malformation, which is also called an oronaso-ocular cleft or paranasal-medial orbitomaxillary cleft. Its etiologic factors are unclear [1]. It occurs sporadically and usually has no familial tendency, syndromic association, or gender dominance [2, 3]. Embryologically, fusion failure of the olfactory placode, the frontonasal process, and the maxillary process lead to the Tessier No. 3 cleft along the naso-optic groove.

The Tessier No. 3 cleft represents diverse clinical presentations, from a simple notch in the nasolabial groove and a coloboma of the lower eyelid to bilateral forms with cranial extensions [4, 5]. Rarely, the lesion directly involves the orbit, which leads to microphthalmia or anophthalmia. True anophthalmia is difficult to distinguish from severe microphthalmia because it can only be diagnosed histologically [6]. Thus, if there is no eyeball involvement, it is called clinical anophthalmia [7]. Generally, the condition of the eyes is a decisive factor in planning surgical sequences for patients with oronaso-ocular clefts.

For bilateral Tessier No. 3 clefts, in particular, no consensus exists for surgical protocols and treatment sequences due to its rarity and complexity. Sesenna reported a two-step closure of a bilateral Tessier No. 3 cleft using Veau III operation, one side by one side, and repeated transnasal canthopexy [8]. Wu reported a staged protocol for a bilateral Tessier No. 3 cleft, which constituted bilateral lip repair first, followed by nasofacial repair with medial canthopexy [9]. Further, Chen introduced the surgical technique of midface rotation advancement in the repair of Tessier No. 3 and No. 4 clefts [10]. In this paper, we present our surgical modality in a patient with severe bilateral Tessier No. 3 cleft.

## Case presentation

**Fig. 1:**
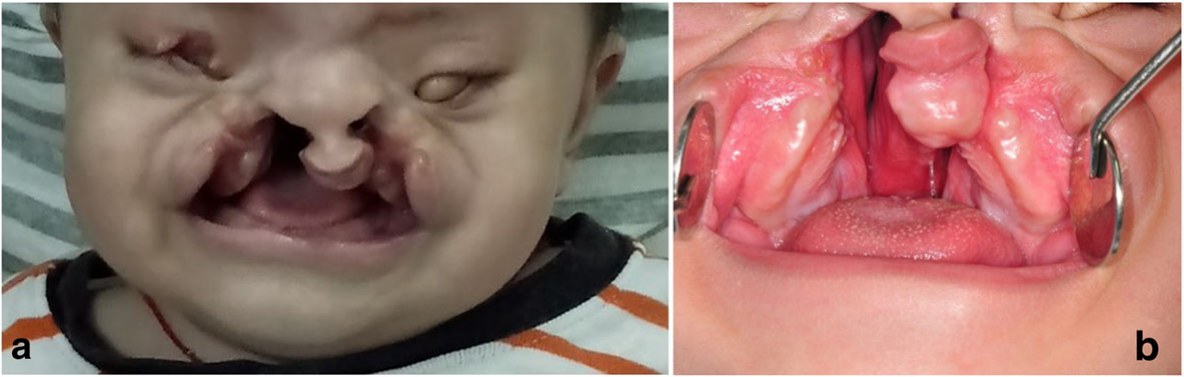
Preoperative clinical photographs. **a** An 8-month-old boy showed bilateral oronaso-ocular clefts with right-sided microphthalmia and left-sided clinical anophthalmia. **b** Intraorally, bilateral severe alveolar clefts and complete palatal cleft were revealed

An 8-month-old Vietnamese baby was referred to Gangneung-Wonju National University Dental Hospital for repair of facial clefts. There was little information about his birth because his parents had abandoned him to a community home. At initial diagnosis, he weighed 8 kg and had no other teratologic lesions. On clinical examination, the soft tissue facial deformities demonstrated asymmetrical bilateral oblique clefts extending from the upper vermilion through the alar grooves up to the lower eyelids. Additionally, inferior displacement of the medial canthi, bilateral medial colobomas (a rectangular coloboma occupying the medial of the right upper eyelid), and no formation of the left eyeball was detected (Fig. 1a). Intraorally, bilateral alveolar and palatal clefts with protruding premaxilla were revealed (Fig. 1b). On ophthalmologic examination, the right eye was in end-stage and had no function due to recurrent retinal detachments.

**Fig. 2:**
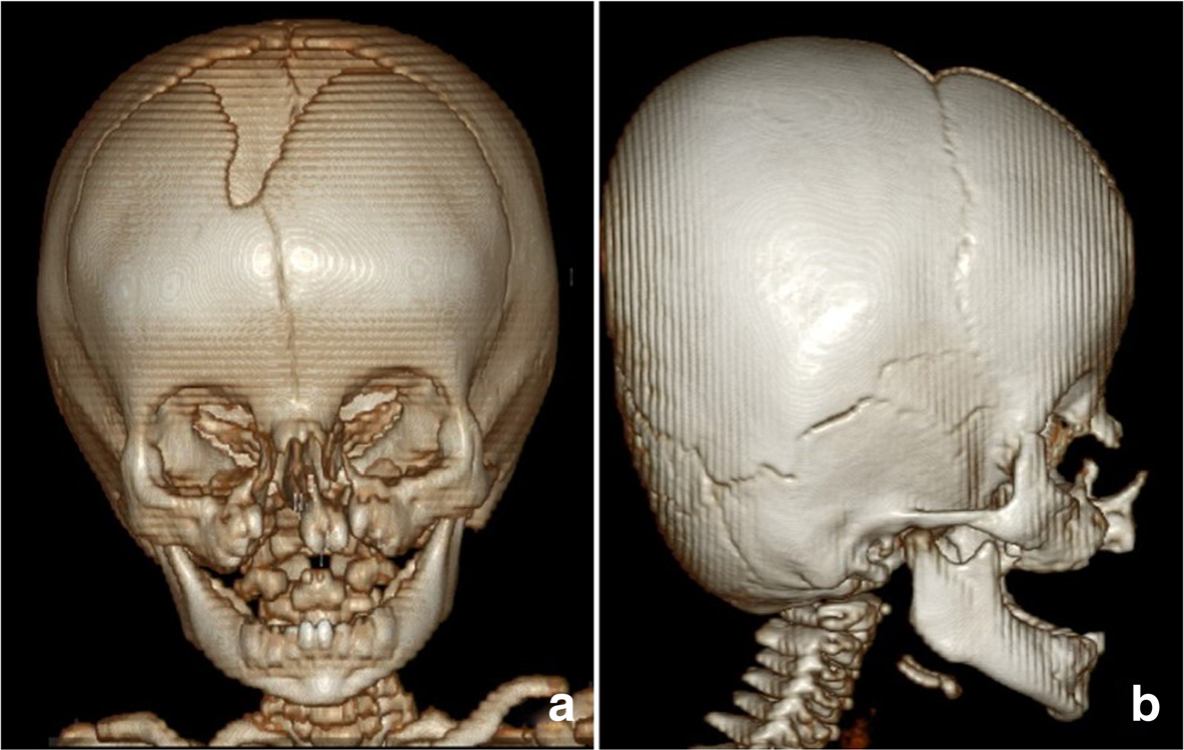
3D CT views. **a** The frontal view demonstrated corresponding bony clefts extended from the alveolus, through the nasomaxillary sutures, and up to the inferomedial walls of the orbits. **b** The lateral view demonstrated a protruding premaxilla

Three-dimensional (3D) computed tomography (CT) and magnetic resonance imaging (MRI) revealed bilateral bony defects from the alveolar bone at the position of the lateral incisor through the lateral wall of the piriform aperture and through the frontal process of the maxilla to the medial orbital wall. Hypoplasia of the maxilla, absence of paranasal sinuses, bony defects of the superior and inferomedial orbital wall on the right side, concave deformity in both medial orbital walls, and medial herniation of orbital contents was detected. No definite signs of dysplasia of the skull base or craniosynostosis were interpreted (Fig. 2). Finally, we diagnosed bilateral oronaso-ocular clefts (Tessier No. 3) extending cranially on the right side (Tessier No. 11), including right-sided microphthalmia without visual acuity, left-sided clinical anophthalmia, and complete bilateral cleft palate.

**Fig. 3:**
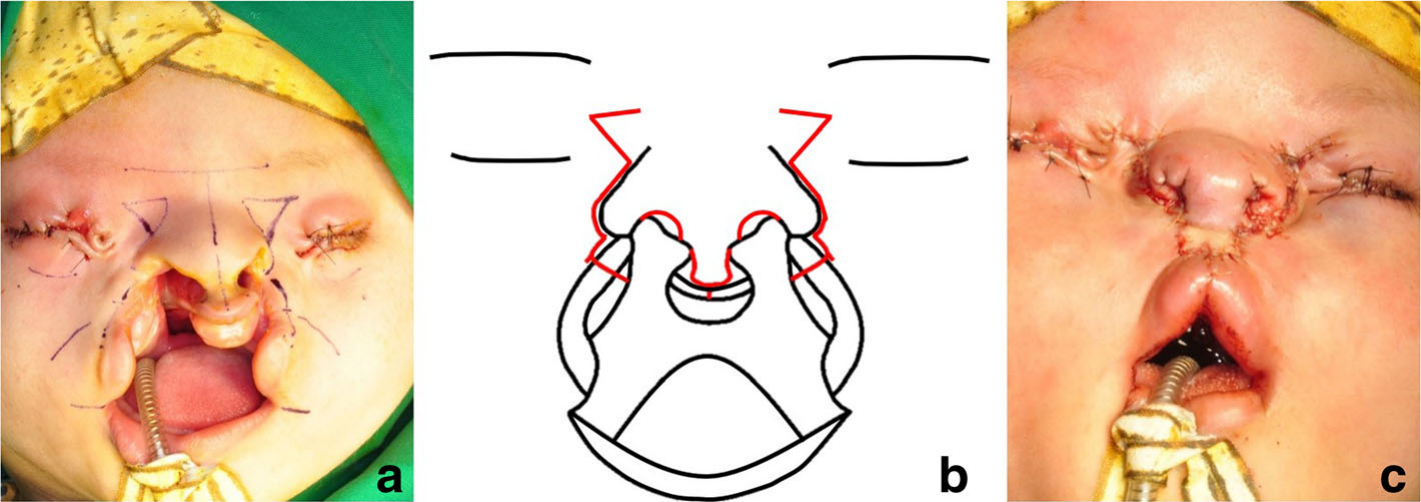
Intraoperative photographs and the schematic design. **a** Incision lines were marked on the prolabium, alar bases, lateral lip segments, and peri-orbital area, after tattooing anatomical landmarks such as peak points for the Cupid’s bow, alar base, nasion, real and expected medial canthus, and mouth corners. **b** The schematic design of surgical incisions. **c** All the sutures are done

In our surgical plan, eyesight preservation was not an issue. Moreover, the lacrimal apparatus was absent on his left side and was beyond repair on his right side. Therefore, we postponed the reconstruction of the eyelid and the infraorbital bony defects. On June 8, 2017, the patient underwent simultaneous repair of the lip and facial clefts with medial canthopexies under general anesthesia. After tattooing anatomic landmarks, such as peak points for the Cupid’s bow, alar base, nasion, real and expected medial canthus, and mouth corners, incision lines were marked on the prolabium, alar bases, lateral lip segments, and peri-orbital areas (Fig. 3a, b). Several ampules of 2% lidocaine containing 1:100,000 epinephrine were injected around the marking line. For the correction of the protruded premaxilla, it was repositioned by ostectomy and set back.

The incision was made from the expected medial canthal point, turning laterally, designing a triangular, medially based upper eyelid flap, and then coming back to the real medial canthal point. The incision was continued through the nasal outline and nasofacial groove to the lateral cleft margin. On the prolabium, the dimension of the philtral flap was 4 mm wide at its superior aspect and 5 mm wide at its inferior aspect, which prepared wider than that of the bilateral cleft lip, for tension-free lip closure. A white skin roll-lip tubercular flap was prepared from the lateral lip segments as in surgical maneuvers for routine bilateral cleft lips.

For reconstruction of the nasal floor, medial and lateral nasal mucosal flaps were prepared. An intraoral vestibular incision and wider subperiosteal dissection were performed to prepare a laterally based cheek flap. To reduce the tension of the cheek flap, the periosteum around the infraorbital neurovascular bundle was incised. The subperiosteal dissection was extended up to the infraorbital margin, which was actually made cleft by the disease. After the dissection of the displaced midfacial musculatures, the medially based nasal flap and alar base were rotated downward for nasal lengthening.

After reconstruction of the nasal floor and intraoral vestibules, the orbicularis oris and facial expression muscles were approximated with the opposite sides or at their anatomic positions for functional reconstruction. Then, the laterally based cheek flap and lateral lip segments were advanced medially. After cutting the inferiorly displaced medial canthal tendon, both side medial canthopexies were performed by suturing the remnants of the medial canthal tendon and the medial edge of the tarsal plate to the paranasal periosteum to lengthen the oculoalar distance. After the lip closure, the medial edge of the cheek flap was approximated with the lateral edge of the nasal flap.

For limited rhinoplasty, the lower lateral cartilages were approximated using two transfixation sutures with 5-0 PDS via marginal nostril incision. Then, the medially based upper eyelid flap was trimmed, transposed, and sutured to the tissue edge of the lower eyelid under the newly formed medial canthus to relieve future scar contractures (Fig. 3c). Finally, 8 units of botulinum toxin type A (Botulax®, Hugel, Seoul, Korea) were injected into four points of the orbicularis oris muscle to relieve possible muscular tension [11, 12]. After the operation, the patient recovered uneventfully.

**Fig. 4:**
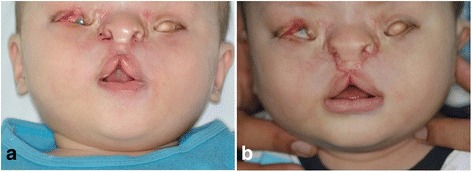
Postoperative follow-up photographs. **a** At suture removal. **b** 3 months after the operation

After 1 week, the skin sutures were removed, and the results were esthetically favorable without unnatural scars (Fig. 4a). Postoperatively, the patient’s appearance seemed to be socially acceptable. However, whistle deformity of the upper vermilion was unavoidable due to soft tissue deficiencies in the paranasal area (Fig. 4b). Redo surgery could resolve the secondary deformity. The patient is planned to undergo palatoplasty, coloboma correction, and ocular prosthetic rehabilitation.

## Conclusions

To our knowledge, severe forms of bilateral Tessier No. 3 clefts have been reported in only a few instances in the English literature. The patient described here presented asymmetric oronaso-ocular clefts with bony involvements, telo-orbitism with right-sided microphthalmia, and left-sided anophthalmia. Our surgical goal was to improve his facial appearance, so we simultaneously corrected the soft tissue clefts, including repositioning the medial canthi. Our main surgical strategies were a downward rotation of the nasal flap, medial advancement of the cheek flap, and the use of an upper eyelid flap. We achieved good results esthetically and functionally by putting the incision line at the borders of anatomic structures and repositioning the displaced facial muscles at their anatomic positions.

## Abbreviation

No.: number
